# A Case of Nonpuerperal Uterine Inversion Caused by Cervical Cancer

**DOI:** 10.1155/2022/1630192

**Published:** 2022-01-31

**Authors:** Daiki Hiratsuka, Takehiro Tsukazaki, Kenbun Sone, Kazuaki Neriishi, Kimihiro Takechi

**Affiliations:** ^1^Department of Obstetrics and Gynecology, The University of Tokyo, 7-3-1 Hongo, Bunkyo-ku, Tokyo, Japan; ^2^Department of Obstetrics and Gynecology, Showa General Hospital, 8-1-1 Hanakoganei, Kodaira-shi, Tokyo, Japan

## Abstract

Uterine inversion is a rare puerperal event in the third stage of labor. Nonpuerperal uterine inversion is even rarer and is mainly caused by uterine fibroids, uterine sarcoma, or endometrial cancer. This is the first report of uterine inversion caused by cervical cancer. A 67-year-old woman presented with a 10 cm pelvic mass. Contrast-enhanced magnetic resonance imaging revealed uterine inversion, which was preoperatively diagnosed to be caused by endometrial cancer and was treated using an extended abdominal hysterectomy. Postoperative histopathological examination revealed that the primary tumor was a squamous cell carcinoma with coexistent high-grade squamous intraepithelial lesions and small-cell neuroendocrine carcinoma. Immunostaining was diffusely positive for p16 and negative for estrogen receptors. The postoperative diagnosis was cervical squamous cell carcinoma. Our observations suggested that cervical carcinoma can cause uterine inversion by invading the corpus.

## 1. Introduction

Uterine inversion, in which the uterine fundus is turned inside out into the uterine cavity, is a rare puerperal event in the third stage of labor and is mainly triggered by umbilical cord traction [1]. In contrast, nonpuerperal uterine inversion is an even rarer event. The primary cause of nonpuerperal uterine inversion is uterine fibroids; however, in some cases, it is also caused by malignant tumors, such as uterine sarcoma [2–9]. It is usually caused by the sudden extrusion of a tumor from the uterus, thinning of the uterine wall, dilatation of the uterine cervix, tumor size, and thickness of the tumor pedicle and tumor attachment site [3–5]. To the best of our knowledge, this is the first case of nonpuerperal uterine inversion caused by cervical cancer.

## 2. Case Presentation

A 67-year-old woman (gravida 2, para 2) was evaluated for a mass in her right breast and was diagnosed with right breast cancer at another center. She was referred to our hospital for the management of a uterine mass that was detected on contrast-enhanced computed tomography (CT) during a search for metastasis. Her medical history was significant for type 2 diabetes mellitus and hyperlipidemia. She had no family history of malignancy.

**Figure 1:**
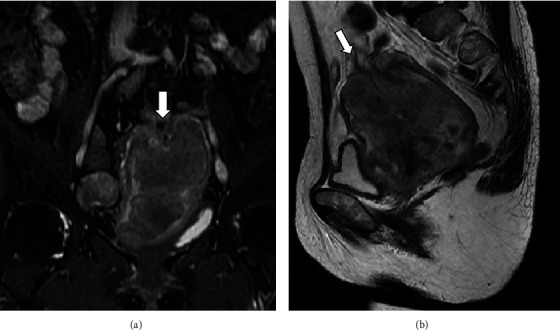
T2-weighted (a) coronal and (b) sagittal MRI images taken before surgery. The arrow shows the inverted fundus of the uterus. The uterus is U-shaped.

On admission, we observed mild vaginal bleeding. A gynecological examination and transvaginal ultrasonography showed a tumor sized 80 × 60 × 40 mm. Biochemical marker analysis showed an elevated hemoglobin A1c level of 8.0%, a neuron-specific enolase level of 28.2 ng/mL, and a squamous cell carcinoma- (SCC-) associated antigen level of 11.2 ng/mL (normal <1.5 ng/mL). Contrast-enhanced magnetic resonance imaging (MRI) revealed an irregular mass in the uterine cavity that had spread to the vaginal cavity, predominantly occupying the uterine corpus, causing uterine inversion (Figures 1(a) and 1(b)). No bladder or rectal infiltration was observed, but the right obturator lymph node was enlarged to a diameter of 2 cm. Parametrium infiltration was unclear, but the cardinal ligaments seemed to be compressed due to the tumor. No disseminated nodules or ascites were observed. In addition, distant metastases were not observed on CT. Tumor tissue biopsy detected SCC with small-cell neuroendocrine carcinoma (SCNEC). Preoperatively, a diagnosis of endometrial cancer was considered. Considering the size and localization of the tumor, extended abdominal hysterectomy was planned.

**Figure 2:**
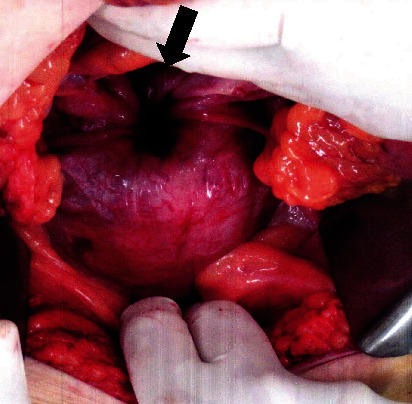
Laparotomy findings. The arrow shows the fundus of the inverted uterus. Bilateral round ligaments and adnexa are also retracted.

On laparotomy, the uterus was inverted. The fundus was depressed, and the bilateral round ligaments and adnexa had retracted (Figure 2). The tumor had replaced the cervix and infiltrated the upper one-third of the posterior vaginal wall. Infiltration into the right parametrium was remarkable, and the right obturator lymph node was enlarged to a diameter of 2 cm. The cardinal ligaments were invaded, but the tumor was not extended to the pelvic wall. Extended abdominal hysterectomy, which included resection of the infiltrated vaginal wall and parametrium as well as the enlarged obturator lymph node, was performed. The total operation time was 5 h and 19 min, and the blood loss was 2,116 mL. The postoperative course was uneventful, and the patient was discharged from our hospital 8 days postoperatively without further complications.

**Figure 3:**
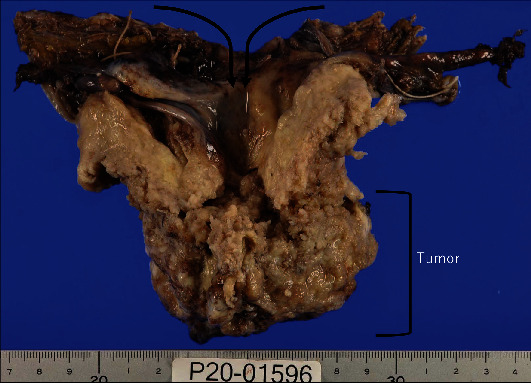
Macroscopic image of the specimen. The uterus is inverted because of the spread of the tumor from the cervix to the corpus. The cervix is replaced with the tumor tissue and cannot be identified. The tumor has invaded the fundus of the uterus, resulting in uterine inversion.

**Figure 4:**
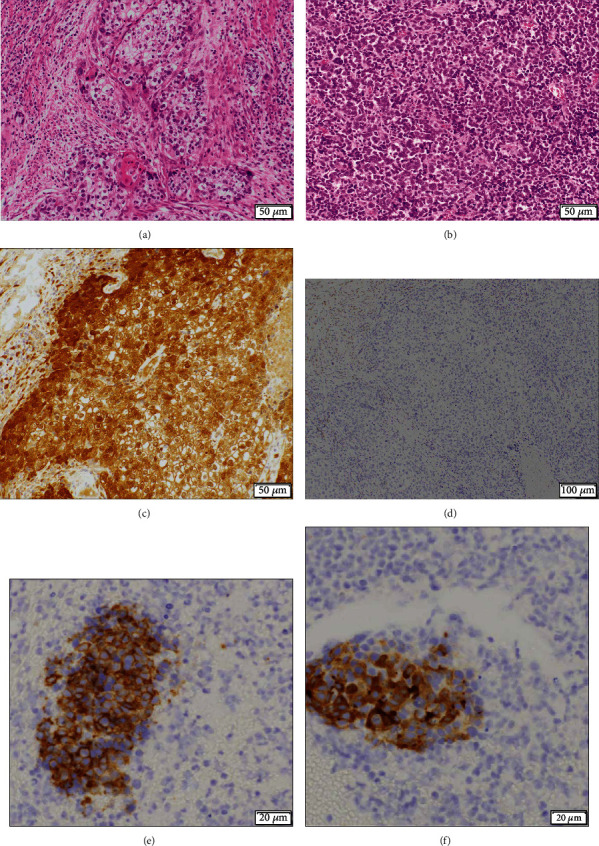
Microscopic findings of the specimen. (a) Hematoxylin and eosin staining of the specimen shows that the main component is squamous cell carcinoma. (b) Hematoxylin and eosin staining of the specimen shows that 20% of the tumor is small-cell neuroendocrine carcinoma. (c) Immunostaining with an anti-p16 antibody is diffusely positive. (d) Immunostaining with an antiestrogen receptor antibody is negative. (e) Immunostaining with an anti-CD56 antibody is positive. (f) Immunostaining with an antisynaptophysin antibody is positive. The white bars indicate 50 *μ*m (a–c), 100 *μ*m (d), and 20 *μ*m (e–f).

The tumor measured 83 × 62 × 40 mm in size, and the cervix was deformed with a mass involving the corpus (Figure 3). The uterine serosa at the fundus was inverted due to traction by the tumor. Vaginal wall infiltration and right parametrial infiltration were observed, and the margin was partially positive. A histopathological examination showed that the main component was SCC, and approximately 20% of the tumor was SCNEC (Figures 4(a) and 4(b)). The epithelial structure of the cervix was partially preserved in the high-grade squamous intraepithelial (HSIL) lesion. HSIL, SCC, and SCNEC were present simultaneously. In addition, immunohistochemically, p16 was diffusely positive, suggesting human papillomavirus infection (Figure 4(c)). Furthermore, the tumor was negative for estrogen receptor, suggesting that the tumor had not originated from the endometrium (Figure 4(d)) and was considered to have formed in the cervix. Immunostaining showed CD56 positivity and synaptophysin positivity (Figures 4(e) and 4(f)). Both the SCC and SCNEC components had metastasized to the submitted right obturator lymph node. The patient was diagnosed with cervical cancer and SCC with SCNEC, Stage IIIC1 (pT2bN1M0).

Surgery for right breast cancer was performed 1 month after the abdominal surgery. Although the cancer was an invasive ductal carcinoma of the breast, it was strongly positive for estrogen receptor and was a separate tumor entity from uterine cancer. The postoperative treatment of the uterine tumor was decided in accordance with the treatment of SCNEC, considering that SCNEC has a high risk for local recurrence and metastasis and occupied 20% of the cervical cancer. Postoperative chemotherapy (etoposide, 100 mg/m^2^ on days 1–3 + cisplatin, 80 mg/m^2^ on day 1: six courses) was performed instead of concurrent chemoradiation therapy (CCRT) [10]. Follow-up examinations, including vaginal examination, monitoring of tumor marker levels, and CT for follow-up of the chemotherapy for cervical cancer, have not shown tumor recurrence until 1 year postoperatively. The patient has had no complications so far.

## 3. Discussion

Uterine inversion is a rare condition in which the uterine fundus is turned inside out into the uterine cavity [1]. Only 200–300 cases of nonpuerperal uterine inversion have been reported so far, which is fewer than the reports on puerperal uterine inversion. Uterine fibroids account for approximately 80% of cases, and the other cases are mainly due to uterine sarcoma [2–9]. The occupying tumor appears similar to a cervical tumor in some cases of nonpuerperal uterine inversion. However, no case associated with cervical cancer has been reported previously [11, 12]. To the best of our knowledge, this is the first reported case of nonpuerperal uterine inversion caused by cervical cancer. Surgery is the standard treatment for nonpuerperal uterine inversion [2], but when the primary cause is cervical cancer, it may sometimes be better to perform CCRT rather than surgery. Therefore, accurate preoperative diagnosis of such cases is important to determine the treatment strategy. Our case report highlights the importance of being aware that cervical cancer could also lead to uterine inversion. MRI can help detect uterine inversion with a U-shaped deformed uterus in the sagittal and coronal sections or a “bull's eye” sign in the axial section [13]. These findings were also observed in this case.

The etiology of uterine inversion depends on the underlying disease, and the influence of individual factors differs on a case-by-case basis. While nonpuerperal uterine inversion is triggered by internal forces, puerperal uterine inversion is triggered by external forces, such as umbilical cord traction and manual removal of the placenta. Internal forces include sudden extrusion of a tumor from the uterus, thinning of the uterine wall, dilatation of the uterine cervix, tumor size, and thickness of the tumor pedicle and tumor attachment site [3–6]. These factors are intricately related and lead to uterine inversion. For example, in the case of submucosal uterine fibroids, the uterine fundus is pulled and weakened by the size and weight of the tumor, and the contraction of the uterus adds further stress. This results in the delivery of the myoma and uterine inversion. In contrast, in the case of endometrial cancer and uterine sarcoma, the uterine fundus is stretched and thinned with tumor growth. Consequently, the tumor growth pulls the uterine fundus toward the corpus, and the uterus contracts to extrude the tumor toward the vagina, leading to uterine inversion. Cervical cancer usually develops laterally and rarely invades into the corpus [14]. However, in this case, the tumor was not limited to the cervix but had invaded the corpus. As the tumor involved the uterine fundus, the uterus was inverted as in endometrial cancer or uterine sarcoma.

One of the risk factors for corpus invasion in cervical cancer is the presence of nonsquamous histology, including SCNEC [10]. SCNEC is reported to account for only 1% of cervical cancers and is a high-grade malignancy with a poor prognosis [10]. In this case, SCNEC occupied 20% of the tumor, which may have contributed to the corpus invasion and uterine inversion. The findings from this case suggest that the uterine inversion was caused by cervical cancer, but the assessment of other similar cases is necessary for establishing the causal association between nonpuerperal uterine inversion and cervical cancer. In summary, to achieve an accurate preoperative diagnosis, cervical cancer must be excluded in the case of uterine inversion if the tumor is a high-grade malignancy invading the corpus.

In this case, we detected an SCC, and the tumor occupied the uterine corpus. Our preoperative diagnosis was endometrial cancer because we considered that the tumor occupied the uterine corpus and that there are a few case reports of primary SCC of the endometrium [15]. In addition, some case reports suggested that nonpuerperal uterine inversion was wrongly attributed to the cervical tumor in the preoperative diagnosis [11, 12]. An accurate preoperative diagnosis is significant because the treatment of cervical cancer is different from other cancers. In the case of cervical cancer, there is a choice between radiation therapy and surgery. Generally, it is true that the treatment for uterine inversion is surgery; however, if the present case was diagnosed as cervical cancer preoperatively, CCRT might have been the first choice of the treatment.

In conclusion, our report represents the first case of nonpuerperal uterine inversion caused by cervical cancer. If the cervical tumor weakens the cervix and involves the fundus, cervical cancer may present as uterine inversion. Upon observing nonpuerperal uterine inversion, clinicians should consider cervical carcinoma as a differential diagnosis to enable accurate preoperative diagnosis and treatment.

## Data Availability

The data used to support the findings of this study are included within the article.

## References

[1] Das P. (1940). Inversion of the uterus. *BJOG*.

[2] Herath R. P., Patabendige M., Rashid M., Wijesinghe P. S. (2020). Nonpuerperal uterine inversion: what the gynaecologists need to know?. *Obstetrics and Gynecology International*.

[3] Rosa Silva B., de Oliveira Meller F., Uggioni M. L. (2018). Non-puerperal uterine inversion: a systematic review. *Gynecologic and Obstetric Investigation*.

[4] Della Corte L., Giampaolino P., Fabozzi A., di Spiezio Sardo A., Bifulco G. (2019). An exceptional uterine inversion in a virgo patient affected by submucosal leiomyoma: case report and review of the literature. *The Journal of Obstetrics and Gynaecology Research*.

[5] Takano K., Ichikawa Y., Tsunoda H., Nishida M. (2001). Uterine inversion caused by uterine sarcoma: a case report. *Japanese Journal of Clinical Oncology*.

[6] Mehra R., Siwatch S., Arora S., Kundu R. (2013). Non-puerperal uterine inversion caused by malignant mixed Mullerian sarcoma. *BML Case Reports*.

[7] de Vries M., Perquin D. A. (2010). Non-puerperal uterine inversion due to submucous myoma in a young woman: a case report. *Journal of Medical Case Reports*.

[8] Salameh A. E. K., Aljaberi L. M., Almarzooqi R. M., Khloof D. R., Abu Ras S. A., Tabanja R. (2019). Non-puerperal uterine inversion associated with adenosarcoma of the uterus: a case report. *Case Reports in Women's Health*.

[9] Al Qahtani N. H. (2018). Chronic incomplete non-puerperal uterine inversion due to huge submucous fibroid: diagnosis and management. *BML Case Reports*.

[10] Tempfer C. B., Tischoff I., Dogan A. (2018). Neuroendocrine carcinoma of the cervix: a systematic review of the literature. *BMC Cancer*.

[11] Kochar S., Nama A., Khajotia S., Suthar N. (2019). Chronic uterine inversion associated with uterine leiomyoma misdiagnosed as cervical fibroid: a case report. *International Journal of Reproduction, Contraception, Obstetrics and Gynecology*.

[12] Umeononihu O. S., Adinma J. I., Obiechina N. J., Eleje G. U., Udegbunam O. I., Mbachu I. I. (2013). Uterine leiomyoma associated non-puerperal uterine inversion misdiagnosed as advanced cervical cancer: a case report. *International Journal of Surgery Case Reports*.

[13] Lewin J. S., Bryan P. J. (1989). MR imaging of uterine inversion. *Journal of Computer Assisted Tomography*.

[14] Matsuo K., Machida H., Blake E. A., Takiuchi T., Mikami M., Roman L. D. (2017). Significance of uterine corpus tumor invasion in early-stage cervical cancer. *European Journal of Surgical Oncology*.

[15] Bogani G., Uccella S., Cromi A. (2014). Primary squamous cell carcinoma of the endometrium in elderly women: a report of four cases. *Aging Clinical and Experimental Research*.

